# *Wolbachia* Utilizes lncRNAs to Activate the Anti-Dengue Toll Pathway and Balance Reactive Oxygen Species Stress in *Aedes aegypti* Through a Competitive Endogenous RNA Network

**DOI:** 10.3389/fcimb.2021.823403

**Published:** 2022-01-21

**Authors:** Wei Mao, Qin Zeng, Lingzhi She, Hao Yuan, Yuying Luo, Renke Wang, Yueting She, Weifeng Wang, Chaojun Wang, Xiaoling Pan

**Affiliations:** ^1^ The Key Laboratory of Model Animals and Stem Cell Biology in Hunan Province, Department of Medical Laboratory Science, Hunan Normal University School of Medicine, Changsha, China; ^2^ The Key Laboratory of Protein Chemistry and Developmental Biology of Fish of Ministry of Education, Hunan Normal University, Changsha, China

**Keywords:** *Wolbachia*, lncRNA, ceRNA, reactive oxygen species, Toll pathway, dengue virus, *Aedes aegypti*

## Abstract

Long non-coding RNAs (lncRNA), a class of RNA molecules without protein coding potential, are more than 200 nucleotides in length and widely present in a variety of species. Although increasing progress in regard to the determination of lncRNA function has been made in vertebrates, *Aedes aegypti* lncRNAs were only identified recently and the functions of few lncRNAs have been annotated so far. Herein, the genome-wide alteration of the lncRNA expression profile trigged by *Wolbachia w*AlbB infection was investigated by comparing *A. aegypti* Aag2 cells and W-Aag2 cells infected with *Wolbachia w*AlbB. Based on lncRNA sequencing, 3035 differentially expressed lncRNAs (DE lncRNAs) in total were identified upon *Wolbachia* infection, which were further validated by quantitative PCR. The constructed co-expression network of DE lncRNAs and mRNAs revealed that *Wolbachia*-induced DE lncRNAs were highly enriched in the oxidative phosphorylation pathway *via trans*-activity, according to the KEGG pathway enrichment analyses. In addition, the established competitive endogenous RNA (ceRNA) network identifies the DE lncRNAs enriched in cellular oxidant detoxification based on GO enrichment analysis. Furthermore, silencing of aae-lnc-7598, the significantly up-regulated lncRNA with the highest fold change induced by *Wolbachia*, caused a significant reduction of antioxidant catalase 1B (*CAT1B*) gene expression as well as the enhancement of mitochondrial reactive oxygen species (ROS) production in living cells. These findings indicate that *Wolbachia* manipulates lncRNA to balance intracellular ROS stress and ensure its endosymbiosis in host *A. aegypti*. Notably, the function assay demonstrated that aae-lnc-0165 suppressed by *Wolbachia* could induce expression of the *REL1* gene, the key regulator of downstream Toll pathway, through the sequence-specific binding of aae-miR-980-5p, which contributes to the activation of Toll pathway. Moreover, the depletion of aae-lnc-0165 caused the suppression of mitochondrial ROS levels in living cells. Our data reveal that *Wolbachia* activates the anti-dengue Toll pathway through a lncRNA-ceRNA pattern. Taken together, our finding suggested that *Wolbachia* utilizes lncRNAs to activate host anti-dengue Toll pathway *via* a ceRNA network. Moreover, *Wolbachia* employs lncRNAs to ensure ROS homeostasis for ROS-based anti-dengue defense through either *trans*-regulation or the ceRNA network. This study identifies novel potential molecular biomarkers for prevention and control of epidemic dengue.

## Introduction

Long non-coding RNAs (lncRNA), a class of RNA molecules without protein coding potential, are more than 200 nucleotides (nt) in length. They are widely present in diverse species, including animals (Barlow and Bartolomei, 2014), plants (Yu et al., 2019), insects (Lopez-Ezquerra et al., 2017; Zhang et al., 2020), prokaryotes (Lejars et al., 2019), and even viruses (Chen et al., 2018; Liu et al., 2020). Based on their genomic location, lncRNAs can be categorized into four types: intergenic lncRNAs (lincRNA), which are transcribed from intergenic regions (Etebari et al., 2016); intronic lncRNAs, transcribed entirely from introns of protein-coding genes (Nakaya et al., 2007); sense lncRNAs, transcribed from the sense strand of protein-coding genes (Ma et al., 2013); and antisense lncRNAs, transcribed from the antisense strand of protein-coding genes (Wang et al., 2018). LncRNAs have been linked to various biological functions through their role in gene expression regulation, which has been reported either on neighboring co-expressed protein-coding genes, namely *cis*-regulation, or on distant protein-coding genes, namely *trans*-regulation (Yan et al., 2017; Kopp and Mendell, 2018; Gil and Ulitsky, 2020). Moreover, lncRNAs function as competing endogenous RNAs (ceRNAs) to compete with mRNAs for interaction with microRNAs (miRNAs) *via* miRNA response elements with reverse complementary binding seed regions, and thus trigger miRNA-mediated target gene regulation (Tay et al., 2014; Thomson and Dinger, 2016; Chan and Tay, 2018; Wang L. et al., 2019). MiRNAs, a class of small single-stranded non-coding RNAs containing about 22 nt, function *via* base-pairing with complementary sequences within mRNA molecules (Krol et al., 2010). The binding of miRNA to non-coding or coding regions of mRNA results in various regulatory effects including gene silencing (Jonas and Izaurralde, 2015), translation activation (Correia de Sousa et al., 2019), and transcriptional and post-transcriptional gene regulation (Fabian et al., 2010). Therefore, the mode of communication with miRNAs *via* ceRNAs make lncRNAs a tool for controlling miRNA functions in diverse biological processes.

Although increasing progress in the elucidation of lncRNA functions has been made in vertebrates (Nitsche and Stadler, 2017; Kopp and Mendell, 2018), functional studies of lncRNAs in *Aedes aegypti* are very limited. *A. aegypti*, a blood-feeding mosquito, is the principal vector responsible for replication and transmission of arboviruses such as dengue virus (DENV) (Pan et al., 2012), Chikungunya virus (CHIKV) (Vega-Rua et al., 2014), and Zika virus (ZIKV) (Aubry et al., 2020). Recently, lncRNAs were identified in *A. aegypti*, and the function of a few lncRNAs has been annotated so far (Etebari et al., 2016; Azlan et al., 2019). Because lncRNAs share low levels of interspecies sequence similarity, they exhibit functional species-specific characteristics (Kopp and Mendell, 2018). It is difficult to predict the potential role of lncRNA in *A. aegypti* based on their similarity with other species. To date, only one study has reported the mutual interaction between lncRNAs and DENV infection as well as the association of differentially expressed lncRNAs with *Wolbachia w*MelPop infection in *A. aegypti* (Etebari et al., 2016). The data revealed the significant potential role of lncRNAs in the *Wolbachia*-induced anti-dengue response in *A. aegypti.*

*Wolbachia*, a gram-negative intracellular symbiotic bacterium, has received considerable attention. Because it confers fascinating characteristics on mosquito vectors, in particular the significant broad-spectrum inhibition of human pathogens including DENV (Pan et al., 2012), ZIKV (Dutra et al., 2016), CHIKV, and malarial parasites (Moreira et al., 2009; Bian et al., 2013). In conferring resistance against DENV invasion, the mosquito Toll signaling pathway activates nuclear factor-kappaB-IkB transcription factor, *REL1*, thus inducing the expression of antimicrobial peptides (Bian et al., 2005; Waterhouse et al., 2007) that are crucial for the anti-dengue immune responses of *A. aegypti* (Xi et al., 2008). In addition, ROS are an essential factor for controlling dengue invasion in *A. aegypti* (Liu et al., 2016). In *A. aegypti* with stable infection of *Wolbachia*, the elevated ROS induced by *Wolbachia w*AlbB participates in ROS-dependent activation of the Toll pathway to enhance resistance to the DENV (Pan et al., 2012). Furthermore, studies have described the role of *Wolbachia w*MelPop miRNAs in DENV inhibition in *A. aegypti* (Hussain et al., 2011). These findings reveal that *Wolbachia* utilizes multiple mechanisms for resistance against the DENV, including those involving ROS, the Toll pathway, miRNA, and lncRNA. However, it is still unknown whether *Wolbachia* manipulates the coordination of ROS, the Toll pathway, miRNA, and lncRNA to weaken dengue transmission ability in *A. aegypti*.

In the present study, 3035 differentially expressed (DE) lncRNAs were identified by comparison between *Wolbachia w*AlbB-infected *A. aegypti* W-Aag2 cells and *A. aegypti* Aag2 cells without *Wolbachia* infection, using deep RNA sequencing and quantitative PCR (qPCR) validation. These lncRNAs share the similar characteristics of low GC content and short length, as previously reported (Etebari et al., 2016). The most abundant DE lncRNAs were intronic and intergenic lncRNA in *A. aegypti*. Furthermore, functional analysis suggested that *Wolbachia*-regulated DE lncRNAs were highly enriched in the oxidative phosphorylation pathway *via trans*-regulation, according to the KEGG database information. Notably, *Wolbachia* induced aae-lnc-7598 elevated antioxidant *CAT1B* gene expression *via trans*-regulation to regulate intracellular ROS stress in *A. aegypti*. Moreover, *Wolbachia* decreased aae-lnc-0165 levels to induce the expression of a key regulator of the Toll pathway downstream, namely the *REL1* gene, *via* aae-miR-980-5p, and thus reduce excessive ROS production. Overall, we determined how *Wolbachia* modulates host lncRNAs to manipulate ROS abundance *via* either *trans*-regulation or through the ceRNA network. Our results demonstrate that *Wolbachia* utilizes *A. aegypti* lncRNAs to activate the anti-dengue Toll pathway through ceRNA network, which contributes to ROS regulation.

## Materials and Methods

### Cell Maintenance and Sampling

The *A. aegypti* Aag2 cells and W-Aag2 cells carrying *w*AlbB *Wolbachia* (Lu et al., 2012) were kindly provided by professor Zhiyong Xi (Department of Microbiology and Molecular Genetics, Michigan State University). These *Aedes* cells were routinely maintained in Schneider’s Drosophila Medium containing L-glutamine, with 10% (v/v) fetal bovine serum and 1% (v/v) penicillin/streptomycin at 26°C following standard procedures described previously (Pan et al., 2018). 293T cells, a highly transfectable derivative of human embryonic kidney 293 cells, were purchased from a properly licensed commercial company, Meilunbio (PWE-HU007, Dalian, China). The 293T cells were grown in Dulbecco’s Modified Eagle Medium (DMEM) with 10% (v/v) fetal bovine serum and 1% (v/v) penicillin/streptomycin at 37°C under 5% (v/v) CO_2_.

For sampling, the Aag2 and W-Aag2 cells were seeded into a 6-well plate at a density of 8 × 10^5^ cells per well. The cell samples for each cell line were collected from day 1 to day 5 post passage with 3 replicates per day; then, for 5 consecutive days, cell mixtures were pooled into three biological replicates for each line. Finally, each biological replicate was divided into aliquots for total RNA extraction.

### RNA Extraction and Quality Control

For sequencing, total RNA was extracted from cell samples using TRIzol reagent (Invitrogen, California, USA), according to the manufacturer’s instructions. Subsequently, RNA integrity and purity were examined on a Bioanalyzer 2100 System (Agilent Technologies, California, USA), using the RNA Pico 6000 Assay Kit (Agilent Technologies, USA). RNA concentration was determined by Qubit 2.0 Fluorometer (Thermo Fisher Scientific, Massachusetts, USA), using the Qubit RNA BR Assay Kit (Invitrogen, USA). The total RNA was used for RNA sequencing and qPCR analysis of lncRNA and mRNA.

For miRNA function assay, miRNA was isolated from cell samples subject to different treatments, using the MiPure Cell/Tissue miRNA Kit (Vazyme, Nanjing, China) according to the manufacturer’s recommendations.

### Construction of lncRNA Libraries and RNA Sequencing

Six lncRNA libraries were constructed for the Aag2 and W-Aag2 cell lines, with three biological replicates per cell line. For each lncRNA library, 2.0 μg of qualified total RNA was initially used for the depletion of ribosomal RNA using a Ribo-Zero rRNA removal kit (Epicentre, Wisconsin, USA), followed by synthesis of cDNA using a NEBNext Ultra Directional RNA Library Prep Kit for Illumina (NEB, USA) as recommended by the manufacturer. Both ends of the cDNA fragments were ligated with specialized adaptors, introducing a unique barcode for each library. Finally, the qualities of six lncRNA libraries were further tested using Qubit 2.0, Agilent Bioanalyzer 2100. Only qualified lncRNA libraries were sequenced *via* Illumina NovaSeq6000 sequencing system on the platform BMKCloud (www.biocloud.net) using a paired-end, 150-bp sequencing strategy.

### Data Processing and lncRNA Identification

Raw sequencing data of fastq format were filtered to obtain clean reads *via* quality assessment of sequencing error rate and GC-content, using in-house Perl scripts. After removal of reads for adapter sequences, reads with high proportion of N (N denotes the unascertained base information), low-quality reads, and clean data with high quality were used for downstream analyses (**Supplementary Table 1**).

The clean reads were mapped on the *A. aegypti* reference genome (GCF_002204515.2) downloaded from Genbank (https://www.ncbi.nlm.nih.gov/genbank/) for transcriptome assembly, using StringTie (Pertea et al., 2015). The assembled transcripts were annotated using the program gffcompare (Pertea and Pertea, 2020). Among the remaining transcripts without annotation, transcripts with length ≥ 200 nt and number of exons ≥ 2 were selected for screening of lncRNA candidates by filtering out the putative protein-coding RNAs, using the following four computational approaches: the coding potential calculator (CPC) (Kong et al., 2007), coding-non-coding index (CNCI) (Sun et al., 2013), PFAM protein families database (http://pfam.xfam.org/), and coding potential assessment tool (CPAT) (Wang et al., 2013). Finally, 25218 potential lncRNAs were identified and used for further classification of intronic lncRNAs, anti-sense lncRNAs, sense lncRNAs, and long intergenic noncoding RNAs (lincRNAs) using the program gffcompare.

### lncRNA Quantification and Differential Expression Analysis

The expression level of lncRNAs was quantified with StringTie (version 1.3.1) software, using the fragments per kilobase of transcript per million fragments mapped (FPKM) value (Trapnell et al., 2010).

Differential expression analysis of lncRNAs between Aag2 and W-Aag2 cells was performed using the DESeq2 (version v1.6.3) package in the R project (Love et al., 2014), based on the false discovery rate (FDR) approach. All *p*-values were adjusted using the Benjamini and Hochberg methods to minimize false discovery rates. LncRNAs with an adjusted FDR<0.05 and absolute value of log_2_(fold change) ≥1 identified by DESeq were assigned as differentially expressed lnRNAs (DE lncRNAs).

### Construction of DE lncRNA-mRNA Co-Expression Network

Based on multiple functional mechanisms for different type of lncRNAs, the lncRNA-mRNA co-expression network was constructed for *csi*-acting lncRNA and *trans*-acting lncRNA, respectively, using Cytoscape software (version 3.7.2) (Shannon et al., 2003). The lncRNAs could be classified as *cis*-acting lncRNAs and *trans*-acting lncRNAs according to the location of the target gene relative to the transcription site of the lncRNA. To examine interactions between *cis*-acting lncRNA and mRNAs, all protein-coding genes near the lncRNAs 100 kb upstream and downstream that were significantly co-expressed with the lncRNA were detected as target genes. The *trans*acting lncRNA and its target mRNA were identified according to the correlation of expression levels for DE lncRNA-mRNA pairs determined by Pearson’s correlation coefficient (PCC) analysis. The threshold of PCC ≥0.9 and *p*-value < 0.01 were considered to indicate significant correlation.

### Construction of DE lncRNA-miRNA-mRNA Networks

Using the miRbase database (http://mirbase.org), the binding relationships of DE lncRNA-miRNA and miRNA-mRNA were obtained by dual-prediction analysis with both miRanda (http://www.microrna.org/microrna/home.do) and TargetScan (http://www.targetscan.org), applying a cutoff criterion of minimum free energy (MFE) ≤ -25 kcal/mol. Only the DE lncRNAs included in all of these interactions were selected for construction of the DE lncRNA-miRNA-mRNA ceRNA network, which was drawn using Cytoscape software (version 3.7.2) (Shannon et al., 2003).

### Functional Enrichment Analysis of lncRNA

These DE lncRNAs were subjected to functional enrichment analysis according to Gene Ontology (GO; http://geneontology.org) functional terms and the Kyoto Encyclopedia of Genes and Genomes pathway database (KEGG, https://www.genome.jp/kegg/), based on their predicted co-expressed genes and target genes *via* ceRNA network. The function of the top 20 significantly enriched DE lncRNA target genes was determined based on the enrichment score of –log_10_(*p*-value).

### cDNA Synthesis of lncRNA, mRNA, and miRNA

Each total RNA sample was divided into two aliquots. One of these RNA aliquots was used for sequencing, and the other RNA aliquot was reverse-transcribed for validation of DE lncRNA and DE mRNA by sequencing using the PrimeScript RT reagent Kit with gDNA Eraser (Takara, Kusatsu, Japan). The miRNA samples were converted into cDNA with a universal DNA adaptor using the Mir-X miRNA First-Strand Synthesis Kit (Takara, Kusatsu, Japan). The cDNA samples were stored at −40°C until determination of miRNA expression.

### Validation of lncRNA and mRNA Expression Level *via* qPCR

The expression levels of DE lncRNAs and mRNA were verified using the CFX96 PCR detection system (Bio-Rad, Berkeley, USA) with the TB Green Advantage qPCR Premix (Takara, Kusatsu, Japan). qPCR conditions were as follows: initial denaturing at 95°C for 3 min, followed by 40 amplification cycles of denaturation at 95°C for 10 s, 55°C for 30 s of annealing, and 72°C for 30 s of extension. The relative expression of each lncRNA and mRNA was analyzed using the 2^-ΔΔCT^ method, with the ribosomal protein S6 (RPS6) gene as the reference gene (Lu et al., 2012). The specific primers for lncRNA are listed in **Supplementary Table 2**. The primers for REL1 (Pan et al., 2012) and CAT1B gene are summarized in **Supplementary Table 3**.

### RNAi of DE lncRNA Using Sequence-Specific Small Interfering RNA (siRNA)

Sequence-specific siRNAs were designed for aae-lnc-0165 and aae-lnc-7598 using webtool (http://sirna.wi.mit.edu/home.php); the negative control (NC) was a random sequence that cannot bind to the genome of *A. aegypti*. The siRNAs were synthesized by Shanghai Gemma Pharmaceutical Co., LTD (**Supplementary Table 4**). At 24 h prior to transfection, cell passage was performed using a 96-well plate with a density of 0.1 × 10^6^ cell per well. When the cells reached 80% confluence, they were transfected with siRNA or NC at concentrations ranging from 300-700 nM using Lipofectamine 2000 reagent. At 48 h, 72 h, and 120 h post transfection, the cell samples were collected, with six biological replicates per group, for further analysis. Transfection efficiency was assessed *via* qPCR.

### Transfection of miRNA Mimics or Inhibitors

In the miRNA gain-of-function assay, the aae-miR-980-5p mimic, a chemically modified double-stranded RNA molecule, and the scrambled control of miRNA mimic were transfected with Lipofectamine 2000 reagent (Invitrogen, California, USA) at a concentration of 200 nM. For miRNA loss-of-function assay, the aae-miR-980-5p inhibitor, a chemically modified reverse complement RNA oligo of mature aae-miR-980-5p, as well as the synthetic negative control (NC) were used to treat cells at a concentration of 200 nM with Lipofectamine 2000 reagent (Invitrogen, California, USA).

In the rescue assay, Aag2 cells were transfected with 500 nM aae-lnc-0165 sequence-specific siRNA and siRNA negative control (NC) using Lipofectamine 2000 reagent (Invitrogen, California, USA). At 48 h post transfection, each cell group was transfected with 200 nM aae-miR-980-5p sequence-specific inhibitor or inhibitor negative control to obtain four treatment groups: aae-lnc-0165 siRNA with aae-miR-980-5p inhibitor, aae-lnc-0165 siRNA with inhibitor NC, siRNA NC with aae-miR-980-5p inhibitor, and siRNA NC with inhibitor NC. Subsequently, cell samples were collected for further analysis at 72 h after the second transfection. The miRNA mimics, inhibitors, mimic NC, and inhibitor NC were all synthesized by Shanghai Gemma Pharmaceutical Co., Ltd. Detailed information is provided in **Supplementary Table 5**.

### Expression Analysis of miRNA Using qPCR

Using TB Green Advantage qPCR Premix (Takara, Kusatsu, Japan), miRNA quantification was conducted with an aae-miR-980-5p sequence-specific forward primer (5 ′ -CGGCCGTTCATTGGGTCATCTAGC-3′) and a universal reverse primer provided in the Mir-X miRNA First-Strand Synthesis Kit (Takara, Kusatsu, Japan), on a CFX96 PCR detection system (Bio-Rad, Berkeley, CA, USA). The thermocycling conditions were as follows: 95°C for 30 sec of denaturation, followed by 40 cycles at 95°C for 5 sec, and 60°C for 30 sec. The expression of miRNAs was normalized by the amount of cDNA templates (100 ng). Finally, the relative quantification of miRNA content was carried out according to the Ct value using the 2^-ΔΔCT^ method.

### Measurement of Mitochondrial ROS in Living Cells

The cells were seeded into 6−well plates at a density of 1×10^6^ cells/well and then transfected with lncRNA siRNA and siRNA NC at a concentration range from 300–700 nM. Following incubation for 48 h at 26°C, MitoSOX Red (Invitrogen, Oregon, USA), a fluorogenic probe specifically targeted to mitochondrial ROS in live cells, was incubated at a final concentration of 3 μM at 37°C for 10 min in dark. Subsequently, the cells were stained with 2 μg/mL DAPI at 26°C for 5 min in dark. The fluorescence signal was visualized under a fluorescence microscope (MingMei MF52-N, Guangzhou, China). Red fluorescence under the Texas Red channel, as visualized with MitoSOX Red, was used as an indicator of the mitochondrial ROS levels. The blue signal under the DAPI channel represented cell nuclei for the purpose of cell counting. There were three replicates for each treatment. Six individual images were taken randomly for each sample, and the MitoSOX Red fluorescence intensities of were normalized to cell number in each image using ImageJ software (Schroeder et al., 2021).

### Construction of Plasmids

Based on the binding regions of aae-lnc-0165 and *REL1* gene predicted using RNAhybrid (https://bibiserv.cebitec.uni-bielefeld.de/rnahybrid/submission.html/) and miRanda, the seed sequences of the binding region were cloned into the unique restriction enzyme cut sites for *Not*I and *Xho*I of the psiCHECK-2 vector located at downstream of the *Renilla* STOP codon at the 3′UTR. The luciferase reporter plasmid (psi-CHECK-2-WT-0165) containing a 26 nt sequence from aae-lnc-0165 with a binding site sequence between aae-lnc-0165 and aae-miR-980-5p, a luciferase reporter plasmid (psi-CHECK-2-MUT-0165) containing the aae-lnc-0165 sequence including a binding site with a four-base mutation, a wild-type *REL1* luciferase reporter plasmid (psi-CHECK-2-WT-REL1) containing a 27-nt sequence of the *REL1* gene with a binding site between the *REL1* gene and aae-miR-980-5p, and a mutant *REL1* luciferase reporter plasmid (psi-CHECK-2-MUT-REL1) in which a sequence from *REL1* with a seven-base mutations was inserted at the binding site was constructed by Sangon Biotech (Shanghai, China) (**Supplementary Figure 1**).

### Dual−Luciferase Reporter Assay

The 293T cells were seeded into 24−well plates at a density of 1×10^5^ cells/well. Subsequently, the cells were co−transfected with 500 ng of plasmid (psi-CHECK-2-WT-0165 or psi-CHECK-2-WT-REL1) and 140 nM mimic reagent (aae-miR-980-5p mimic or mimic negative control) using Lipofectamine 2000 reagent (Invitrogen, California, USA). At 48 h post transfection, the relative luciferase activities were detected using a dual−luciferase reporter assay system (Promega, Madison, USA) and the firefly activity was used as an internal reference for normalization. The measurement was performed based on ten biological replicates, with three technical replicates for each sample.

### Statistical Analysis

The PCC was calculated to determine the correlation between lncRNA and mRNA. Fisher’s exact test was used to detect the significantly enriched KEGG pathways using ClusterProfiler (Yu et al., 2012). The *p*-value was further used for multiple hypothesis test correction to calculate the q-value. The smaller the q-value, the more significant the enrichment of the KEGG pathway. One-way ANOVA was used for pairwise comparison among multiple groups. Student’s t test was used for pairwise comparison between two groups. A *p*-value lower than 0.05 was considered to indicate statistical significance. The experimental results are expressed as mean ± standard deviation (Mean ± SD). All data are analyzed with GraphPad prism v8.0.2.263.

## Results

### *Wolbachia w*AlbB Induces Differential Expression of lncRNA in *Aedes aegypti*

**Figure 1:**
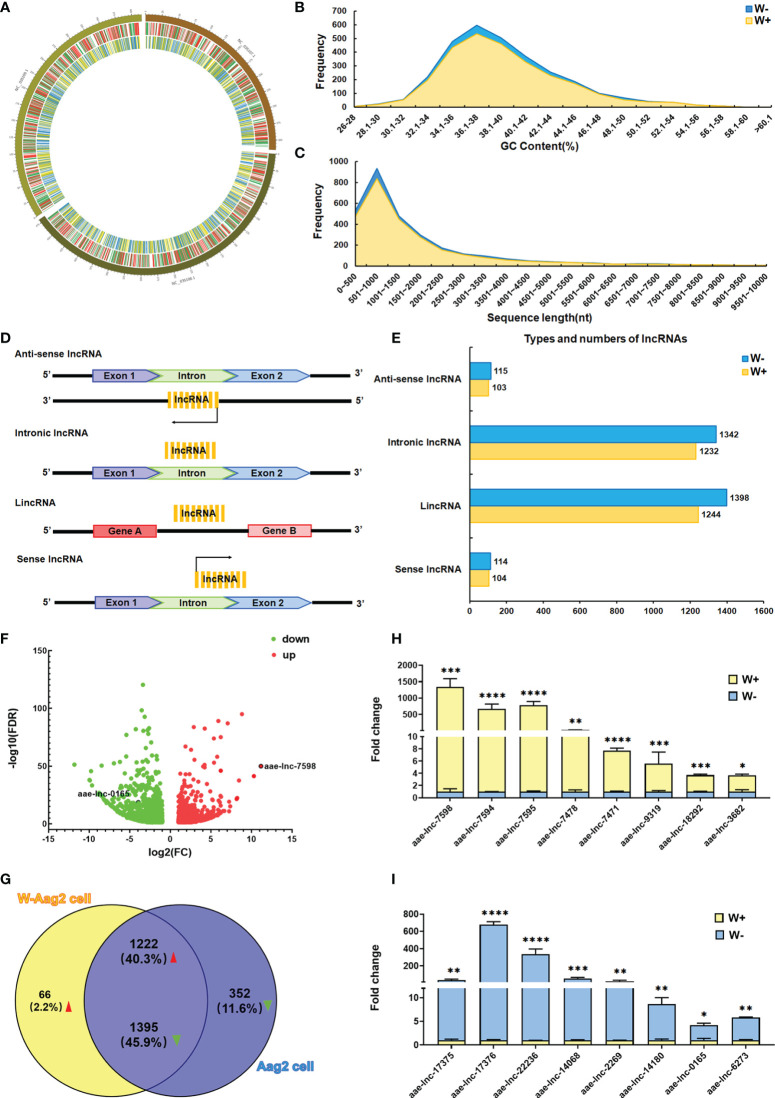
*Wolbachia w*AlbB induces differential expression of lncRNA in *A. aegypti*. **(A)** Distribution of differentially expressed (DE) lncRNAs and mRNAs on a chromosome map. The outer circle shows the chromosome map of *A. aegypti*, the middle circle indicates the distribution of DE mRNAs on the chromosomes of *A. aegypti*, and the inner circle shows the distribution of DE lncRNAs on the chromosomes of *A. aegypti*. The red and green lines in the circle represent upregulated genes and downregulated genes, respectively. Yellow and blue lines represent up-regulated lncRNAs and down-regulated lncRNAs, respectively. **(B)** The GC content of DE lncRNAs identified in *A. aegypti*. **(C)** The length distribution of identified DE lncRNAs. **(D)** Schematic diagram of the origin and type of lncRNAs. **(E)** Types and numbers of DE lncRNAs; the lncRNAs are classified into four types. For each type of lncRNA, the number of lncRNAs per cell line are presented in different colors. **(F)** Volcano maps of 3035 DE lncRNAs; the red dots indicate upregulated lncRNAs, green dots indicate downregulated lncRNAs. **(G)** Venn diagram of the 3035 DE lncRNAs in *A. aegypti*. qPCR validation of significantly down-regulated lncRNAs **(H)** and up-regulated lncRNAs **(I)** in lncRNA sequencing; ****P < 0.0001; ***P < 0.001; **P < 0.01, *P < 0.05.

The transcription levels of long intergenic non-coding RNAs (lincRNAs) have been demonstrated to be regulated by *Wolbachia w*MelPop in *A. aegypti* (Etebari et al., 2016). These findings suggest a high possibility that *Wolbachia w*AlbB could also trigger alterations in lncRNA expression in *A. aegypti*. To test this hypothesis, genome-wide lncRNA screening was conducted to compare *A. aegypti* Aag2 cells (W- cells) and W-Aag2 cells (W+ cells) carrying *Wolbachia w*AlbB. Because *A. aegypti* lncRNAs display a highly temporal expression pattern (Azlan et al., 2019; Farley et al., 2021), the total RNA collected from day 1 to day 5 post passage was pooled as three biological replicates per cell line. This ensured the richness of generated lncRNA libraries. Using the lncRNA libraries, the lncRNA sequencing yielded more than 61 million double-ended clean reads with at least 94.69% Q30 bases for each cell sample on the Illumina NovaSeq 6000 sequencing system (**Supplementary Table 1**), after filtering out the adapter sequences and low-quality reads. These high-quality clean reads were used for sequence alignment with the *A. aegypti* reference genome (GCF_002204515.2) using HISAT, followed by annotation and comparison with the reference genome using StringTie and Gffcompare. Based on identification using four different bioinformatic tools, namely CPC, CNCI, PFAM, and CPAT, 16297 and 16892 reliable lncRNA transcripts were confirmed in W- and W+ cells, using all of the bioinformatic tools, respectively. Furthermore, the relative expression level of lncRNAs was determined for differential expression analysis, based on RNA sequencing data and FPKM values, using String, Gffcompare, and DESeq2. A total of 3035 differentially expressed lncRNAs (DE lncRNAs) was identified, applying criteria of FDR <0.05 and |FC|≥2 (**Figure 1**).

To clarify whether *Wolbachia* manipulates the characteristics of DE lncRNAs in *A. aegypti*, the distribution, GC content, length distribution, and lncRNA classification were analyzed for 3035 differentially expressed lncRNAs. Based on chromosome loci whose sequences overlapped with those of the lncRNAs, *w*AlbB-induced DE lncRNAs were located on three *A. aegypti* chromosomes (NC_035107.1, NC_035108.1, and NC_035109.1) (**Figure 1A**). The mean GC content of DE lncRNAs was 39.21% in both Aag2 and W-Aag2 cells (**Figure 1B**). The length of DE lncRNAs ranged from 200 to 3000 nt, which accounted for 85.42% and 77.40% of DE lncRNAs in Aag2 and W-Aag2 cells, respectively (**Figure 1C**). According to the location of lncRNAs on the genome, the DE lncRNAs were classified into four categories (**Figure 1D**) (Ma et al., 2013): lincRNAs (47.45%), intronic lncRNAs (44.88%), sense lncRNAs (3.79%), and anti-sense lncRNAs (3.89%) (**Figure 1E**). The major type of *A. aegypti* lncRNAs were lincRNAs and intronic lncRNAs in Aag2 and W-Aag2 cells. These data suggest that rather than modifying the characteristics of lncRNAs (i.e. their distribution, GC content, length distribution, and classification), *Wolbachia w*AlbB caused the alteration of lncRNA expression.

To better understand the alteration of lncRNA expression in *A. aegypti* following *Wolbachia w*AlbB infection, the 3035 DE lncRNAs were sorted into 1288 up-regulated lncRNAs and 1747 down-regulated lncRNAs (**Figure 1F**). Notably, 66 up-regulated DE lncRNAs and 352 down-regulated DE lncRNAs were only expressed in W-Aag2 cells and Aag2 cells, respectively (**Figure 1G**). To further verify the differential expression of lncRNAs in response to *Wolbachia w*AlbB infection, the expression profile for top ten induced or suppressed DE lncRNAs was validated *via* qPCR (**Figures 1H, I**). Sixteen DE lncRNAs were identified by both lncRNA sequencing and qPCR. Taken together, 3035 DE lncRNAs were identified based on sequencing data and comprehensive bioinformatic analysis.

### aae-lnc-7598 Induced by *Wolbachia* Mediates *trans*-Regulation of the Antioxidant Catalase 1B Gene to Balance Intracellular ROS Stress in *A. aegypti*

**Figure 2:**
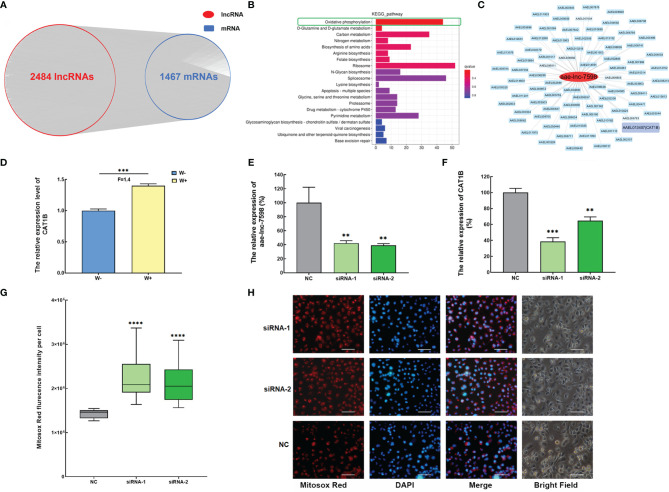
*Wolbachia* induces aae-lnc-7598 to mediate *trans*-regulation of the antioxidant catalase 1B gene to balance intracellular ROS stress in *A. aegypti*. **(A)** The co-expression network of DE lncRNA-mRNA *via trans*-regulation mechanism. **(B)** KEGG pathway enrichment analyses of DE lncRNA *via trans*-acting. **(C)** The co-expression network of aae-lnc-7598. The red circle represents aae-lnc-7598, while the blue rectangle represents mRNA. Among them, the purple rectangular represents the *CAT1B* gene. **(D)** The relative expression level of *CAT1B* in W-Aag2 (W+) and Aag2 cells (W-). **(E)** The relative expression of aae-lnc-7598 at 72-hour post transfection. **(F)** The relative expression of *CAT1B* gene at 72-hour post transfection. **(G)** The fluorescence intensity of mitochondrial ROS per cell at 72-hour post-transfection using the Mitosox Red probe. The bar represents the range of maximum and minimum value, and the horizontal shows the median. NC represents W-Aag2 cells transfected with siRNA control; siRNA-1 and siRNA-2 represent the aae-lnc-7598 depleted W-Aag2 cells obtained by transfection with two individual siRNAs of aae-lnc-7598. **(H)** Representative image of siRNA-transfected W-Aag2 cells at ×40 magnification. The mitochondrial ROS and nucleus were stained with Mitosox Red probe (red) and DAPI (blue). The white line indicates 50 micrometers; ****P < 0.0001; ***P < 0.001; **P < 0.01. F means fold change.

To explore the function of *Wolbachia*-induced DE lncRNAs, the spatiotemporal co-expression of the DE lncRNA-mRNA network was established on the basis of lncRNA mediated *trans*- and *cis*-regulation of mRNA. Out of 3035 DE lncRNAs, 2484 showed a potential *trans*-regulatory role against 1467 mRNAs in *A. aegypti* in PCC analysis, with PCC ≥0.9 and *p*-value < 0.01 (**Figure 2A**). Subsequently, the functional analysis of 2484 *trans*-acting lncRNAs was performed based on their 1467 potential co-expressed genes using KEGG pathway enrichment analyses. Most of the top 20 pathways were associated with *A. aegypti* metabolism. In particular, the top significant enriched pathway was the oxidative phosphorylation pathway (**Figure 2B**). These data reveal that DE lncRNAs induced by *Wolbachia* mainly mediate the *trans*-regulation of energy metabolism of *A. aegypti*, particularly the oxidative phosphorylation pathway.

In the oxidative phosphorylation pathway, ROS, an essential player involved in restricting DENV infection in mosquitoes (Liu et al., 2016; Zhu et al., 2019), was strongly induced by *Wolbachia* in *A. aegypti* (Pan et al., 2012). We speculate that *Wolbachia* might utilize lncRNA to regulate ROS. To test this hypothesis, the significantly induced aae-lnc-7598 with highest fold change based on RNA sequencing and qPCR results was chosen for further functional research (**Figures 1F, H** **)**. Further, aae-lnc-7598, a 281-nt lincRNA, showed interaction with *CAT1B* gene, the only gene associated with ROS synthesis in the constructed co-expression network (**Figure 2C**). The *CAT1B* gene, an important antioxidant-enzyme-encoding gene, was significantly up-regulated in *Wolbachia w*AlbB-infected W-Aag2 cells (**Figure 2D**). To investigate the predicted *trans*-regulation of the *CAT1B* gene by aae-lnc-7598, the loss-of-functional assay was performed in W-Aag2 cells. The depletion of aae-lnc-7598 was conducted *via* the transfection of sequence-specific siRNAs, which was compared to the cell group treated with the random-sequence siRNA control. A greater than 57% knockdown efficiency was detected in cells treated with aae-lnc-7598-specific siRNA at 72 h post transfection, which caused the significant suppression of *CAT1B* expression by 61% and 35% (siRNA-1 and siRNA-2, respectively) (**Figures 2E, F** **)**. These results reveal that *Wolbachia w*AlbB induces elevation of the expression of antioxidant *CAT1B* by aae-lnc-7598. It has been reported *A. aegypti-*induced antioxidant combats the damaging effects of ROS caused by *Wolbachia w*AlbB infection (Pan et al., 2012), and the effect of aae-lnc-7598 on intracellular ROS was evaluated using a mitochondrial ROS-specific fluorescent probe. Strikingly, the suppression of *CAT1B via* silencing of aae-lnc-7598 led to a 1.56-fold (*P*<0.00001) and 1.49-fold (*P*<0.00001) increase in the mitochondrial ROS level in siRNA-1- and siRNA-2-treated W+ cells, respectively (**Figures 2G, H** **)**. Taken together, these data suggest that *Wolbachia w*AlbB induces up-regulation of the antioxidant *CAT1B* gene by aae-lnc-7598 *via trans*-regulation and thus balances intracellular ROS stress caused by *Wolbachia* infection.

### aae-lnc-0165 Down-Regulated by *Wolbachia* Induces *REL1 via* aae-miR-980-5p to Activate the Toll Pathway and Regulate ROS

**Figure 3:**
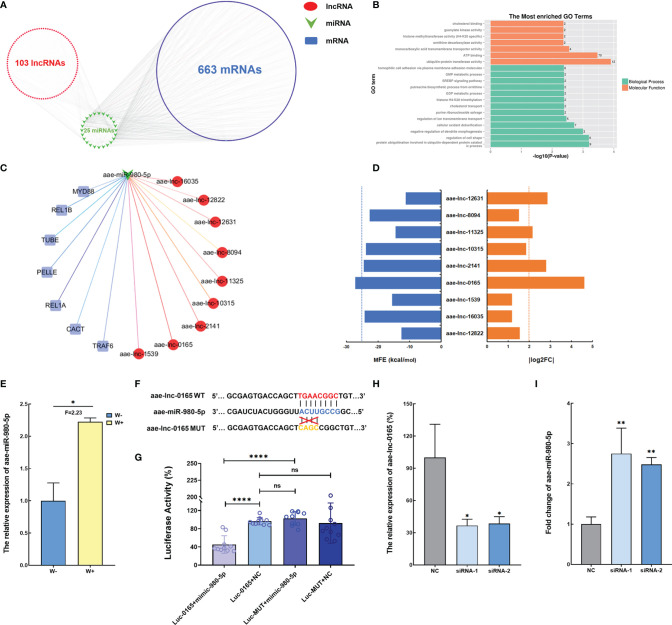
*Wolbachia* reduces aae-lnc-0165 to induce aae-miR-980-5p **(A)** The ceRNA network of DE lncRNA-miRNA-mRNA. **(B)** GO enrichment analyses of target genes for DE lncRNAs. **(C)** The ceRNA network based on the Toll pathway regulator genes and DE lncRNAs. Each red circle represents a lncRNA; the green arrow represents miRNAs, while the blue rectangular represents immune genes. **(D)** The MFE value and absolute value of log2 (fold change) for DE lncRNAs associated with the Toll pathway. The blue dotted line indicates MFE threshold of -25kcal/mol and the orange dotted line indicates that the cutoff value for $\log_{2}\left(fold\;change\right)$ is 2. **(E)** The relative expression level of aae-miR-980-5p in W-Aag2 (W+) and Aag2 (W-) cells. **(F)** Schematic diagram of the target binding sites between aae-miR-980-5p and aae-lnc-0165 predicted by RNAhybrid software. **(G)** Dual luciferase reporter assay indicates the binding relationship between aae-lnc-0165 and aae-miR-980-5p. Luc-0165+mimic-980-5p represents the 293T cells co-transfected with psi-CHECK-2-WT-0165 and aae-miR-980-5p mimic; Luc-0165+NC indicates the 293T cells co-transfected with psi-CHECK-2-WT-0165 and mimic control; Luc-MUT+mimic-980-5p represents the 293T cells co-transfected with psi-CHECK-2-MUT-0165 and aae-miR-980-5p mimic; Luc-MUT+NC indicates the 293T cells co-transfected with psi-CHECK-2-MUT-0165 and mimic control; n=10. **(H)** The relative expression of aae-lnc-0165 at 48-hour post transfection. **(I)** The expression of aae-miR-980-5p at 72-hour post transfection; ****P < 0.0001; **P < 0.01; *P < 0.05; ns means no significant difference. F means fold change.

ROS acts as an essential factor in the activation of the Toll pathway in *A. aegypti* mosquito infected with *Wolbachia* (Pan et al., 2012), and function assay revealed that ROS levels are manipulated by *Wolbachia via* aae-lnc-7598. Therefore, we hypothesize that the lncRNAs regulated by *Wolbachia* might communicate with the Toll pathway. Consequently, the direct and indirect modulation of Toll regulatory genes by *Wolbachia*-induced DE lncRNA was examined. Initially, indirect interaction was established based on DE lncRNA-miRNA pairs and miRNA-mRNA pairs identified using the TargetScan and miRanda bioinformatic analysis. The ceRNA network constructed indicated that 103 DE lncRNAs might indirectly regulate 663 mRNAs *via* 25 miRNAs, which are associated with cellular oxidant detoxification based on GO enrichment analysis (**Figures 3A, B** **)**. Among the ceRNA network associated with Toll pathway regulating genes, the potential binding relationship between aae-lnc-0165, a 1959-nt lincRNA down-regulated by *Wolbachia* with the highest fold change, and aae-miR-980-5p, a miRNA that is significantly induced by *Wolbachia*, was selected for further functional research based on the threshold of MFE < -25 kcal/mol and | log_2_(fold change) | > 2 (**Figures 3C–E**).

To further determine the binding between aae-lnc-0165 and aae-miR-980-5p, RNAhybrid software was used for prediction of the binding seed region in the interaction between aae-lnc-0165 and aae-miR-980-5p. The aae-lnc-0165 sequence fragment with the binding seed region was cloned into the psiCHECK-2 vector downstream of the *Renilla* luciferase reporter gene, generating the plasmid psi-CHECK-2-WT-0165. As a control, plasmid psi-CHECK-2-MUT-0165 was established, which contains a mutation within the binding seed region (**Figure 3F** and **Supplementary Figure 1**). To avoid the interference caused by endogenous miRNAs from *A. aegypti* cells, the 293T cell line, a commonly used human embryonic kidney (HEK) cell line in transfection assays, was employed to validate the aae-lnc-0165 target site *via* a dual-luciferase reporter assay. Luciferase activity was significantly reduced in 293T cells co-transfected with psi-CHECK-2-WT-0165 and aae-miR-980-5p mimic relative to the control group co-transfected with psi-CHECK-2-WT-0165 and mimic control. No change in luciferase activity was detected in cells transfected either aae-miR-980-5p mimic or mimic control in response to psi-CHECK-2-MUT-0165 (**Figure 3G**). In short, these data strongly suggest that aae-lnc-0165 could bind directly to aae-miR-980-5p.

To confirm that aae-lnc-0165 serves as an endogenous molecular sponge for aae-miR-980-5p, two siRNAs for aae-lnc-0165 were constructed to silence aae-lnc-0165 in Aag2 cells. A knockdown efficiency of more than 61% was achieved at 48 h post transfection in each siRNA-treated cell group relative to the control group treated with the siRNA control (**Figure 3H**). The expression of aae-miR-980-5p was significantly enhanced, by 2.75-fold and 2.48-fold, at 72 h post transfection of siRNA-1 or siRNA-2, respectively (**Figure 3I**). Furthermore, the increase of aae-miR-980-5p was consistently detected at 120 h post transfection of siRNA-1 or siRNA-2 in Aag2 cell (**Supplementary Figure 2**). These data suggest that *Wolbachia* suppressed aae-lnc-0165 to increase the expression of aae-miR-980-5p in *A. aegypti*.

**Figure 4:**
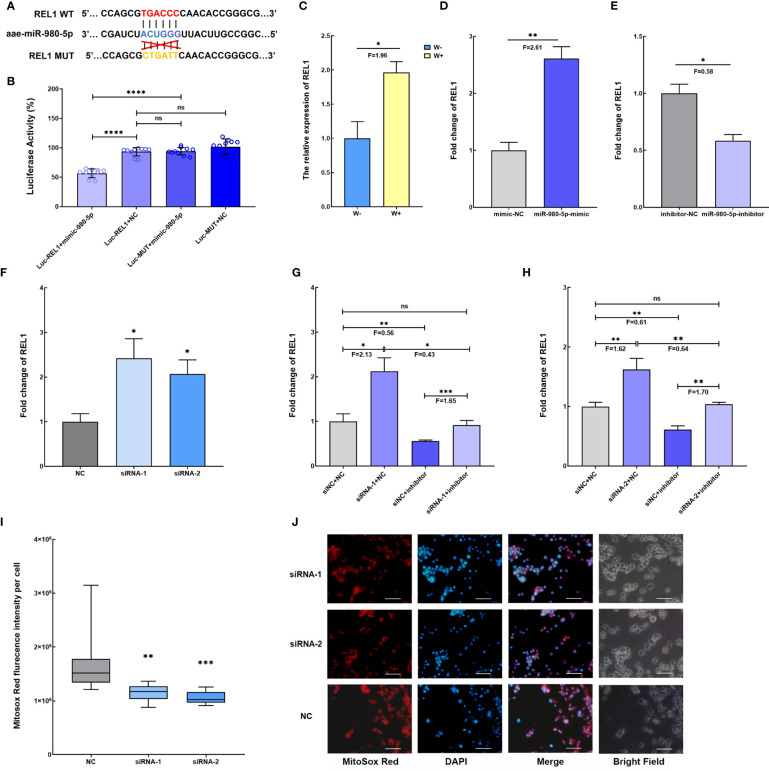
*Wolbachia*-induced aae-miR-980-5p *via* aae-lnc-0165 increases *REL1* expression, contributing to the regulation of intracellular ROS stress. **(A)** Schematic diagram of the predicted target binding sites between aae-miR-980-5p and *REL1* by RNAhybrid. **(B)** Dual luciferase reporter assay confirms the binding sites of aae-miR-980-5p and *REL1*. Luc-REL1+mimic-980-5p represents the co-transfection of psi-CHECK-2-WT-REL1 and aae-miR-980-5p mimic; Luc-REL1+NC represents the co-transfection of psi-CHECK-2-WT-REL1 and mimic control; Luc-MUT+mimic-980-5p represents the co-transfection of psi-CHECK-2-MUT-REL1 and aae-miR-980-5p mimic; Luc-MUT+NC represents co-transfection of psi-CHECK-2-MUT-REL1 and mimic control. **(C)** The relative expression level of *REL1* in W-Aag2 (W+) and Aag2 (W-) cells. **(D)** The gain-of-function assay shows that increasing aae-miR-980-5p induces the expression of *REL1* at 48 h post-transfection. **(E)** The loss-of-function assay indicates that silencing of aae-miR-980-5p reduces the expression of *REL1* at 48 h post-transfection. **(F)** The fold change of *REL1* expression at 120 h post transfection with siRNA for aae-lnc-0165. Validation of the regulatory relationship for aae-lnc-0165, aae-miR-980-5p, and *REL1 via* rescue assay using two sequence specific siRNA-1 **(G)** and siRNA-2 **(H)** for aae-lnc-0165. siNC+NC represents the cell group treated with siRNA control and inhibitor control; siRNA-1/-2+inhibitor represents the cell group treated with siRNA-1/-2 for aae-lnc-0165 and aae-miR-980-5p inhibitor; siNC + inhibitor represents the cell group treated with siRNA control and aae-miR-980-5p inhibitor; siRNA-1/-2 +NC represents the cell group treated with siRNA-1/-2 and inhibitor control. **(I)** The fluorescence intensity of mitochondrial ROS per cell at 72 h post transfection using the Mitosox Red probe. The bar represents the range of maximum and minimum value, and the horizontal shows the median. NC represents Aag2 cells transfected with siRNA control. siRNA-1 and siRNA-2 indicates the aae-lnc-0165-depleted W-Aag2 cells by transfection of two distinct siRNAs for aae-lnc-0165. **(H)** The representative image of siRNA-transfected Aag2 cells at ×40 magnification. The mitochondrial ROS and nucleus were stained with the Mitosox Red probe (red) and DAPI (blue). The white line indicates 50 micrometers; ****P < 0.0001; ***P < 0.001; **P < 0.01; *P < 0.05; ns means no significant difference. F means fold change.

Based on the ceRNA network, aae-miR-980-5p might target the *REL1* gene, a downstream regulator gene of the Toll pathway (**Figure 3C**). To clarify the interaction between *Wolbachia*-induced aae-miR-980-5p and *REL1*, the dual−luciferase reporter assay was performed in 293T cells using a psi-CHECK-2-WT-REL1 construct containing the predicted binding seed region, and psi-CHECK-2-MUT-REL1 containing the mutated binding seed region (**Figure 4A** and **Supplementary Figure 1** **)**. The luciferase activity in cells transfected with psi-CHECK-2-WT-REL1 was significantly decreased in respond to aae-miR-980-5p mimic compared with that in cells transfected with psi-CHECK-2-MUT-REL1. There was no significant difference in luciferase activity in control cell groups either co-transfected with psi-CHECK-2-WT-REL1 and mimic control or co-transfected with psi-CHECK-2-MUT-REL1 and mimic control (**Figure 4B**). These data confirm the binding site between aae-miR-980-5p and *REL1* mRNA.

A function assay was conducted to evaluate the role of aae-miR-980-5p in *REL1* gene expression. *REL1* expression was induced by *Wolbachia w*AlbB in W-Aag2 cells (**Figure 4C**), which is consistent with observations in *Wolbachia w*AlbB-infected *A. aegypti* mosquitoes in previous research (Bian et al., 2005). A gain-of-function assay was performed in Aag2 cells to mimic over-expression of aae-miR-980-5p *via* sequence-specific mimics, which caused increased *REL1* expression (F=2.61, *P*<0.01) relative to mimic control-treated cells at 72 h post transfection (**Figure 4D**). In contrast, a loss-of-function assay conducted in W-Aag2 cells to suppress the abundance of aae-miR-980-5p using a sequence-specific inhibitor led to decreased (F=0.58, *P*<0.05) *REL1* expression compared with that in inhibitor control-treated cells at 72 h post transfection (**Figure 4E**). These data establish the positive regulation of *REL1* expression by aae-miR-980-5p. Taken together, the findings show that *Wolbachia*-induced aae-miR-980-5p enhances the expression of *REL1* in *A. aegypti*.

To examine the regulatory effects of aae-lnc-0165 on *REL1* expression *via* aae-miR-980-5p, the depletion of aae-lnc-0165 was conducted using silencer siRNAs in Aag2 cells. At 120 h post transfection, a significant increase in *REL1* expression was observed, by 2.42-fold and 2.07-fold, in siRNA-1- and siRNA-2-treated cells, respectively (**Figure 4F**). In summary, *Wolbachia*-reduced aae-lnc-0165 contributes to *REL1* over-expression in *A. aegypti*.

To further validate whether aae-lnc-0165-mediated up-regulation of *REL1* expression is modulated through aae-miR-980-5p, we performed a rescue assay to compare expression of *REL1* after silencing aae-lnc-0165, aae-miR-980-5p, and both in Aag2 cells. Although *REL1* expression was increased upon aae-lnc-0165 silencing, when the down-regulation of aae-lnc-0165 was followed by the depletion of aae-miR-980-5p, the expression of *REL1* was restored to a level similar to the control group with treatment of siRNA control and inhibitor control (**Figures 4G, H** **)**. In conclusion, *Wolbachia*-suppressed aae-lnc-0165 could elevate the expression of *REL1 via* aae-miR-980-5p. Considering *REL1* is the key transcriptional regulator of the mosquito Toll immune pathway (Bian et al., 2005), our data show that *Wolbachia* uses lncRNA to activate the Toll pathway in *A. aegypti*.

The essential role of Toll pathway activation is the expression of antioxidant genes to counteract the damaging effects of ROS in *A. aegypti*. We speculate that *Wolbachia*-suppressed aae-lnc-0165 might influence ROS levels through the ceRNA network in *A. aegypti*. To address the question, the intracellular ROS level was examined in Aag2 cells with aae-lnc-0165 silencing, and was found to be significantly reduced, by 1.48-fold and 1.60-fold, in siRNA-1- and siRNA-2-transfected cells, respectively, compared with that in siRNA control-treated cells (**Figures 4I, J** **)**. Thus, the results demonstrate that *Wolbachia*-suppressed aae-lnc-0165 could decrease ROS levels *via* the ceRNA network in *A. aegypti*.

## Discussion

Using a computational pipeline, thousands of lncRNAs have been identified based on RNA-seq data of *A. aegypti* from public databases (Azlan et al., 2019). Although the differential expression of lncRNA has been associated with *Wolbachia w*MelPop infection in *A. aegypti*, few systematic analyses have characterized *Wolbachia* regulated-lncRNAs in *A. aegypti* (Etebari et al., 2016). Here, we aimed to decipher how *Wolbachia w*AlbB regulates *A. aegypti* lncRNAs. To this end, identification and characterization of DE lncRNAs were performed to compare *A. aegypti* Aag2 cell and W-Aag2 cell stably infected with *Wolbachia w*AlbB, using genome-wide lncRNA sequencing. A total of 3035 DE lncRNAs induced by *Wolbachia w*AlbB were identified; these shared the same characteristics with lncRNAs from other species, including low GC content, and short length (Azlan et al., 2021a). In agreement with a previous classification of lncRNAs in *A. aegypti* mosquitoes and cells (Azlan et al., 2019), our data consistently showed that lincRNAs and intronic lncRNAs accounted for the top two types of lncRNAs with the largest proportion of DE lncRNAs regulated by *Wolbachia w*AlbB in *A. aegypti* cells. These findings suggest that *Wolbachia* triggers alteration of lncRNA expression levels rather than modifying the characteristics of length, GC content, and type on genomic location in *A. aegypti*. In addition, considering that 66 lncRNAs were only expressed in W-Aag2 cells and *Wolbachia* produces small non-coding RNAs function in cross-kingdom communication between the endosymbiont and host *A. aegypti* (Mayoral et al., 2014), *Wolbachia* might encode its own lncRNAs: this is an interesting hypothesis that we aim to explore in future research.

Currently, there are no experimental data illustrating the specific mechanisms underlying the functions of lncRNA in direct or indirect interaction in *A. aegypti*. In particular, functional annotation shows that *Wolbachia*-regulated lncRNAs are only just beginning to be explored. Here, the function of aae-lnc-7598 with the highest fold change triggered by *Wolbachia* was linked to the regulation of ROS, a proven essential inhibitor of the DENV, *via* the *trans*-regulation of the antioxidant enzyme-encoding *CAT1B* gene of *A. aegypti*. Moreover, *Wolbachia*-suppressed aae-lnc-0165 manipulates ROS abundance *via* the ceRNA network. Notably, our findings provide the first evidence of links between *A. aegypti* lncRNAs and ROS *via* both direct and indirect regulation. Consistent with our observations, ROS-related lncRNAs have been documented in vertebrates, in which lncRNAs play a negative or positive role in the oxidation and antioxidant system (Wang X. et al., 2019). Based on our previous studies, *Wolbachia*-induced ROS and antioxidants have been identified in *A. aegypti*. The antioxidant regulatory feedback plays an important role in counteracting the potential damaging effects of ROS on infected host cells and permitting *Wolbachia* to maintain persistent infection in *A. aegypti* (Pan et al., 2012). Taken together, these data suggest that when *Wolbachia* induces high ROS in the host mosquito, it also utilizes differentially expressed lncRNAs to exert protective roles in balancing oxidative stress in *A. aegypti*. These functional lncRNAs provide a novel specific biomarker to better understand how *Wolbachia* manipulates host ROS homeostasis and redox balance, to ensure a stable and suitable microenvironment for ROS-based anti-dengue defense.

*A. aegypti* lncRNAs has been reported to be involved in the host-arbovirus interaction (Etebari et al., 2016). It is reported that lncRNAs are differentially expressed in mosquitoes upon ZIKV and DENV infection (Azlan et al., 2021b). Additionally, the silencing of a lincRNA candidate (lincRNA_1317) has been shown to cause increased viral replication in *A. aegypti* (Etebari et al., 2016). However, the potential molecular mechanisms underlying the anti-dengue response is unknown. In the present research, we demonstrated that *Wolbachia*-suppressed aae-lnc-0165 could increase the expression of *REL1*, the Toll pathway NF-kappaB-IkB transcription factor in *A. aegypti*, *via* binding with aae-miR-980-5p. Thus, the activated Toll pathway contributes to decreasing ROS stress. The correlation between the lncRNA-based ceRNA network and activation of Toll pathway revealed that multiple factors such as ROS, lncRNA, and miRNA are utilized by *Wolbachia* to act in concert in the activation of the Toll pathway. Because the Toll pathway plays a central role in blocking DENV infection in *A. aegypti*, our data imply that *Wolbachia*-regulated lncRNAs serve as a potential protector in eliminating DENV *via* activation of the Toll pathway. We aim to further evaluate the effect of the strength of cross-talk between lncRNAs and the Toll pathway on *Wolbachia*-induced dengue interference in the *A. aegypti* mosquito and cells in our future research. We demonstrate here the novel mechanism of lncRNA-mediated activation of the Toll pathway by *Wolbachia w*AlbB in *A. aegypti*, and our findings provide potentially useful insights to support *Wolbachia*-boosted host immunity.

## Conclusion

In this study, genome-wide lncRNA sequencing showed that *Wolbachia w*AlbB induces 3035 differentially expressed lncRNAs with similar characteristics of low GC content, short length, and classification. The function assay revealed that *Wolbachia* utilizes lncRNA to manipulate host ROS homeostasis and redox balance *via* direct and indirect regulation. Moreover, our data show that *Wolbachia*-regulated lncRNAs serve as a novel regulator in the activation of the Toll pathway *via REL1*, suggesting the prominent potential of lncRNAs as a novel biomarker for dengue prevention and control.

## Data Availability Statement

The datasets presented in this study can be found in online repositories. The names of the repository/repositories and accession number(s) can be found below: NCBI Sequence Read Archive (http://www.ncbi.nlm.nih.gov/sra/) under the NCBI Bioprojects PRJNA722598 and PRJNA789930; with accession numbers SRR14264039, SRR14264040, SRR14264041, SRR17266930, SRR17266931 and SRR17266932.

## Ethics Statement

The animal study was reviewed and approved by the Ethics Committee on Laboratory Animal Care of Hunan Normal University (No.2017-114, No.2018-023, and No. 2021-391).

## Author Contributions

Written informed consent to participate in this study was provided by the participants’ legal guardians/next of kin. WM and XP formulated the hypothesis and designed the project. XP funded the project. WW and CW performed cell culture and sampling. RW and YS conducted functional analysis. HY and YL detected the miRNA, lncRNA, and mRNA expression by qPCR. QZ and LS performed the dual−luciferase reporter assay. WM and QZ performed the function assay and ROS measurement. WM, QZ, LS, and XP prepared and wrote the manuscript. WM, QZ, and XP revised and approved the final manuscript. All authors contributed to the article and approved the submitted version.

## Funding

This study was financially supported by the National Natural Science Foundation of China under grants 81873967 and 81702036; the Natural Science Foundation of Hunan Province, China under grants 2019JJ20013 and 2018RS3068; grants from Hunan Normal University (20CSY104, 20CSY10, 21CSY059, 21CSY058, and KF2021018). YL was supported by a Hunan Normal University fellowship.

## Conflict of Interest

The authors declare that the research was conducted in the absence of any commercial or financial relationships that could be construed as a potential conflict of interest.

## Publisher’s Note

All claims expressed in this article are solely those of the authors and do not necessarily represent those of their affiliated organizations, or those of the publisher, the editors and the reviewers. Any product that may be evaluated in this article, or claim that may be made by its manufacturer, is not guaranteed or endorsed by the publisher.

## Supplementary Material

Supplementary Figure 1 Schematic representation of the construct psi-CHECK-2 plasmids used in dual luciferase reporter assay. **(A)** psi-CHECK-2-WT-0165 contains a 26-nt aae-lnc-0165 sequence that includes the predicted binding sites with aae-miR-980-5p marked in red. **(B)** psi-CHECK-2-MUT-0165 contains a 26-nt aae-lnc-0165 sequence that includes the mutated binding sites with aae-miR-980-5p. **(C)** psi-CHECK-2-WT-REL1 contains a 27-nt sequence from the coding region of *REL1* gene which includes the predicted binding sites with aae-miR-980-5p. **(D)** psi-CHECK-2-MUT-REL1 contains a 27-nt sequence from the coding region of *REL1* gene which include the mutated binding sites with aae-miR-980-5p.

Supplementary Figure 2 Fold change of aae-miR-980-5p at 120-hour post transfection; ***P* < 0.01, **P* < 0.05.
