# Supplementary figures and images for: Wolbachia Utilizes lncRNAs to Activate the Anti-Dengue Toll Pathway and Balance Reactive Oxygen Species Stress in Aedes aegypti Through a Competitive Endogenous RNA Network
Source: Front Cell Infect Microbiol. 2022 Jan 21;11:823403. doi: 10.3389/fcimb.2021.823403 (PMC8814319; doi:10.3389/fcimb.2021.823403)

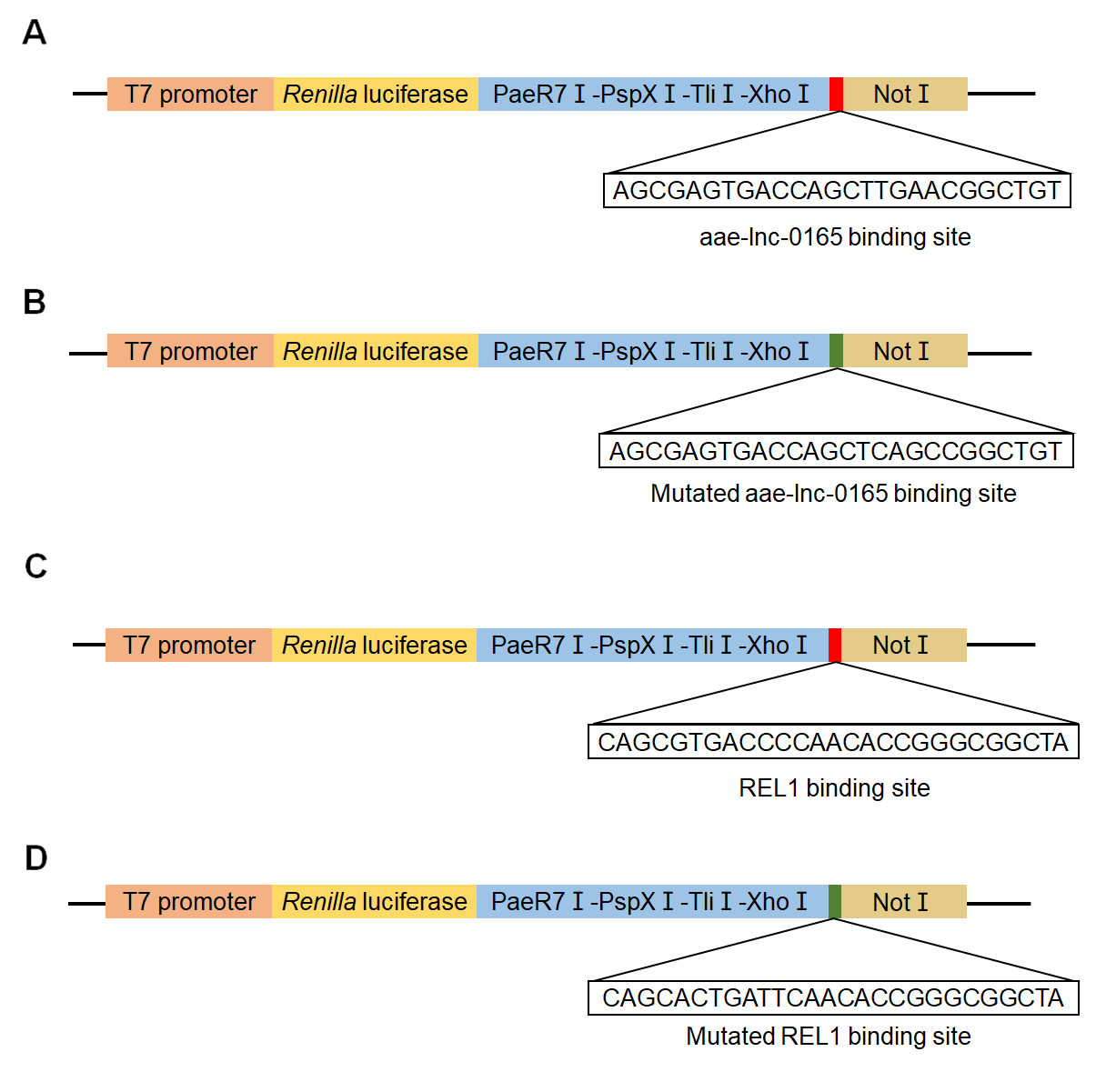

Supplement: Supplementary Figure 1 — Schematic representation of the construct psi-CHECK-2 plasmids used in dual luciferase reporter assay. (A) psi-CHECK-2-WT-0165 contains a 26-nt aae-lnc-0165 sequence that includes the predicted binding sites with aae-miR-980-5p marked in red. (B) psi-CHECK-2-MUT-0165 contains a 26-nt aae-lnc-0165 sequence that includes the mutated binding sites with aae-miR-980-5p. (C) psi-CHECK-2-WT-REL1 contains a 27-nt sequence from the coding region of REL1 gene which includes the predicted binding sites with aae-miR-980-5p. (D) psi-CHECK-2-MUT-REL1 contains a 27-nt sequence from the coding region of REL1 gene which include the mutated binding sites with aae-miR-980-5p. [file Image_1.tif]

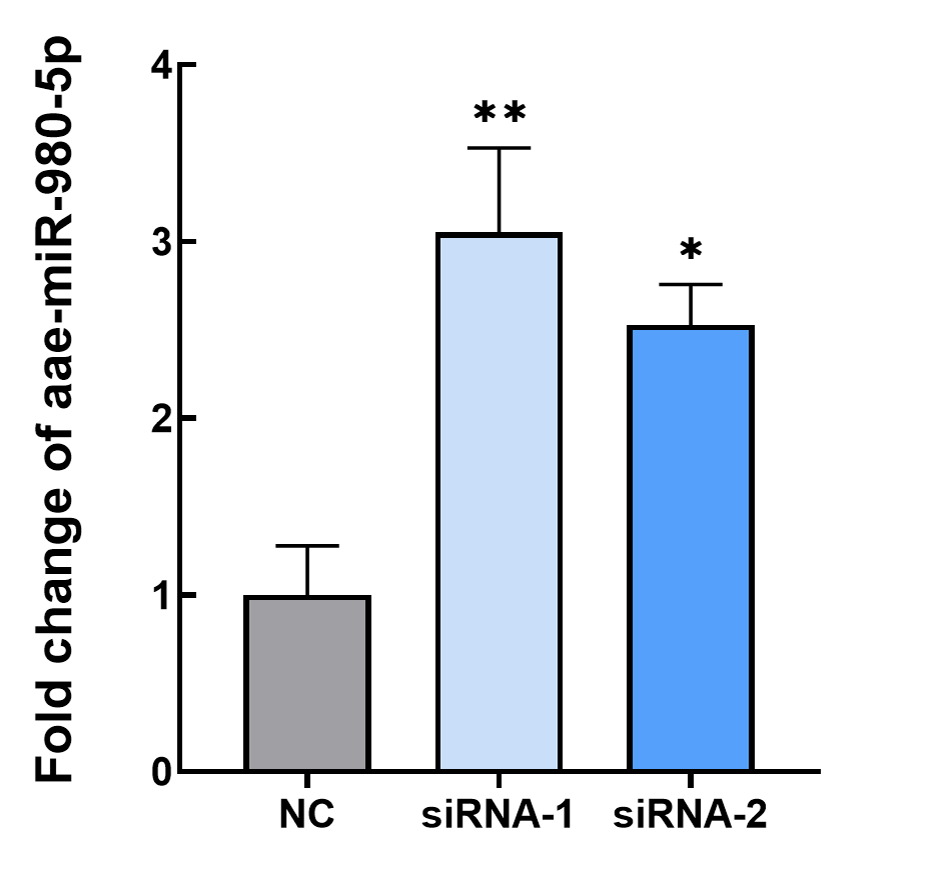

Supplement: Supplementary Figure 2 — Fold change of aae-miR-980-5p at 120-hour post transfection; **P < 0.01, *P < 0.05. [file Image_2.tif]
