# Supplementary material for: Wolbachia Utilizes lncRNAs to Activate the Anti-Dengue Toll Pathway and Balance Reactive Oxygen Species Stress in Aedes aegypti Through a Competitive Endogenous RNA Network
Source: Front Cell Infect Microbiol. 2022 Jan 21;11:823403. doi: 10.3389/fcimb.2021.823403 (PMC8814319; doi:10.3389/fcimb.2021.823403)
Supplement: Supplementary file 3 [file Table_1.docx]

Supplementary Material

**Supplementary Table 1. Summary of sequencing information**

| **Sample ID** | **Read Sum** | **Base Sum** | **GC (%)** | **Q20 (%)** | **Q30 (%)** |
| --- | --- | --- | --- | --- | --- |
| Aag2 cell-S1 | 65,842,186 | 19,187,767,604 | 46.7 | 98.34 | 94.69 |
| Aag2 cell -S2 | 75,348,994 | 21,986,383,310 | 46.75 | 98.47 | 95.04 |
| Aag2 cell -S3 | 61,336,957 | 17,746,444,496 | 47.05 | 98.43 | 94.99 |
| W-Aag2 cell-S1 | 68,490,028 | 20,051,457,978 | 45.3 | 98.5 | 95.23 |
| W-Aag2 cell-S2 | 69,949,174 | 20,427,647,102 | 44.92 | 98.49 | 95.13 |
| W-Aag2 cell-S3 | 77,682,099 | 22,811,977,270 | 44.79 | 98.39 | 94.9 |

Sample ID: sample name; Read Sum: The total number of pair-end Reads in Clean Data; Base Sum: Clean Data Total Base Number; GC (%): The proportion of G and C bases in the total bases of Clean Data; Q20(%): The proportion of bases with a Clean Data quality value greater than or equal to Q20 (base call accuracy of 99 %); Q30(%): The proportion of bases with a Clean Data quality value greater than or equal to Q30 (base call accuracy of 99.9%).
