# Supplementary material for: Wolbachia Utilizes lncRNAs to Activate the Anti-Dengue Toll Pathway and Balance Reactive Oxygen Species Stress in Aedes aegypti Through a Competitive Endogenous RNA Network
Source: Front Cell Infect Microbiol. 2022 Jan 21;11:823403. doi: 10.3389/fcimb.2021.823403 (PMC8814319; doi:10.3389/fcimb.2021.823403)
Supplement: Supplementary file 4 [file Table_2.docx]

Supplementary Material

**Supplementary Table 2. The primers used for validation of DE lncRNAs**

| **lncRNA ID** | **lncRNA sequence 5’-3’** |
| --- | --- |
| aae-lnc-7598-(Forward) | TCGGACGAGAACTGGTGTTT |
| aae-lnc-7598-(Reverse) | TTGCATGCGTCCAATTTTAG |
| aae-lnc-0165-(Forward) | CGTGGAGCCCTGACTGAAAC |
| aae-lnc-0165-(Reverse) | GGTGCATGTCAGAAATCTTAC |
| aae-lnc-17375-(Forward) | AGCTTTGCAACCCTTCTCAA |
| aae-lnc-17375-(Reverse) | TTGTCGTCCTTGAACTGTCG |
| aae-lnc-17376-(Forward) | CTACGCCCATCAACGGTACT |
| aae-lnc-17376-(Reverse) | TGGATATCTCGCTCAGGGCT |
| aae-lnc-22236-(Forward) | CGAGTGGAGCAAAGATTGCG |
| aae-lnc-22236-(Reverse) | GTCGTGGGGTGATAAACGGT |
| aae-lnc-14068-(Forward) | TCTGGTTTGTCACGAGTGCT |
| aae-lnc-14068-(Reverse) | TGTAATACGGCCTCAACCGC |
| aae-lnc-7594-(Forward) | CCATCATTTGGAGGCACTTT |
| aae-lnc-7594-(Reverse) | TTTGCATCCTGAGGGTGATT |
| aae-lnc-7595-(Forward) | ATCGGACGAGAACTGGTGTT |
| aae-lnc-7595-(Reverse) | AAAGTGCCTCCAAATGATGG |
| aae-lnc-2269-(Forward) | CGACCAACTAGCGCAAGACT |
| aae-lnc-2269-(Reverse) | GTCGTCCTCATCGGAGTGTC |
| aae-lnc-6273-(Forward) | CTCATCTTCGGTAGGCAGGT |
| aae-lnc-6273-(Reverse) | TCAGCGTGCGTGATTCATGT |
| aae-lnc-14180-(Forward) | GGTGGCAACTTAGCAAACCG |
| aae-lnc-14180-(Reverse) | GAAGTACGGGCACTGCTGTT |
| aae-lnc-9319-(Forward) | AGGTCGGCCAAAGATCCAAG |
| aae-lnc-9319-(Reverse) | GCGCCAAGATTTCGAATCGG |
| aae-lnc-18292-(Forward) | TGCGTAAGCAGTTTGCATCC |
| aae-lnc-18292-(Reverse) | TGGGTTGAAACCTGAGCCTT |
| aae-lnc-3682-(Forward) | TAGCCGCCTTGTTCCTAGTG |
| aae-lnc-3682-(Reverse) | TTAATGCGCGGGTACCGTAG |
| aae-lnc-7478-(Forward) | CATTCCGCAAATCGTCGTCC |
| aae-lnc-7478-(Reverse) | GGCGCAGGCATTTTATGTGT |
| aae-lnc-7471-(Forward) | TCATGGTTCCGTTGCTCCTC |
| aae-lnc-7471-(Reverse) | TTTCTCTGCTCAGGGATGGC |
