# Supplementary material for: Wolbachia Utilizes lncRNAs to Activate the Anti-Dengue Toll Pathway and Balance Reactive Oxygen Species Stress in Aedes aegypti Through a Competitive Endogenous RNA Network
Source: Front Cell Infect Microbiol. 2022 Jan 21;11:823403. doi: 10.3389/fcimb.2021.823403 (PMC8814319; doi:10.3389/fcimb.2021.823403)
Supplement: Supplementary file 5 [file Table_3.docx]

Supplementary Material

**Supplementary Table 3. The primers used for measurement of mRNA expression**

| **Gene ID** | **Primer Name** | **Primer Sequence5’-3’** |
| --- | --- | --- |
| AAEL000032 | RPS6-(Forward) | GAAGTTGAACGTATCGTTTC |
| AAEL000032 | RPS6-(Reverse) | GAGATGGTCAGCGGTGATTT |
| AAEL013407 | CAT1B-(Forward) | AACATCATTTCCGCCTATCG |
| AAEL013407 | CAT1B-(Reverse) | GATACCATTTCCGCCGTAGA |
| AAEL007696 | REL1-(Forward) | TGGTGGTGGTGTCCTGCGTAAC |
| AAEL007696 | REL1-(Reverse) | CTGCCTGGCGTGACCCGTATCC |
