# Supplementary material for: Wolbachia Utilizes lncRNAs to Activate the Anti-Dengue Toll Pathway and Balance Reactive Oxygen Species Stress in Aedes aegypti Through a Competitive Endogenous RNA Network
Source: Front Cell Infect Microbiol. 2022 Jan 21;11:823403. doi: 10.3389/fcimb.2021.823403 (PMC8814319; doi:10.3389/fcimb.2021.823403)
Supplement: Supplementary file 6 [file Table_4.docx]

Supplementary Material

**Supplementary Table 4. lncRNA specific siRNAs used in loss-of-function assay**

| **lncRNA ID** | **Reagent Name** | **siRNA Sequence 5’-3’** |
| --- | --- | --- |
| aae-lnc-7598  aae-lnc-7598 | 7598-siRNA-1 | GGACGCAUGCAACGAUUAUTT |
|  |  | ACGUGACACGUUCGGAGAATT |
|  | 7598-siRNA-2 | UCGAAUCCCGUUCCGGCAATT |
|  |  | UUGCCGGAACGGGAUUCGATT |
| aae-lnc-0165  aae-lnc-0165 | 0165-siRNA-1 | GGAGCCCUGACUGAAACAATT |
|  |  | UUGUUUCAGUCAGGGCUCCTT |
|  | 0165-siRNA-2 | GGUGCAUGUCAGAAAUCUUATT |
|  |  | UAAGAUUUCUGACAUGCACCTT |
| / | siRNA Negative Control | UUCUCCGAACGUGUCACGUTT |
|  |  | ACGUGACACGUUCGGAGAATT |
