# Supplementary material for: Wolbachia Utilizes lncRNAs to Activate the Anti-Dengue Toll Pathway and Balance Reactive Oxygen Species Stress in Aedes aegypti Through a Competitive Endogenous RNA Network
Source: Front Cell Infect Microbiol. 2022 Jan 21;11:823403. doi: 10.3389/fcimb.2021.823403 (PMC8814319; doi:10.3389/fcimb.2021.823403)
Supplement: Supplementary file 7 [file Table_5.docx]

Supplementary Material

**Supplementary Table 5. Information of mimic and inhibitor for aae-miR-980-5p**

| **Reagent Name** | **Sequence 5’-3’** |
| --- | --- |
| aae-miR-980-5p mimics | CGGCCGUUCAUUGGGUCAUCUAGC |
|  | UAGAUGACCCAAUGAACGGCCGUU |
| mimics Negative Control | UUCUCCGAACGUGUCACGUTT |
|  | ACGUGACACGUUCGGAGAATT |
| aae-miR-980-5p inhibitor | GCUAGAUGACCCAAUGAACGGCCG |
| inhibitor Negative Control | CAGUACUUUUGUGUAGUACAA |
